# The Need for a Concert of Cytogenomic Methods in Chromosomic Research and Diagnostics

**DOI:** 10.3390/genes16050533

**Published:** 2025-04-29

**Authors:** Yiping Wang, Thomas Liehr

**Affiliations:** 1 Jena University Hospital, Friedrich Schiller University, Institute of Human Genetics, Am Klinikum 1, D-07747 Jena, Germany; wangyiping_lamgc@163.com; 2 Jinze Group, Beijing Jinze Medical Laboratory, Building A, Beijing 102206, China; 3 National Engineering Center for Protein Drugs, Building 1, Beijing 102206, China

**Keywords:** cytogenomics, chromosomic, cytogenetics, fluorescence in situ hybridization, chromosome microarray, Sanger sequencing, next-generation sequencing, optical genome mapping

## Abstract

This review focuses on the experimental methods and technologies of cytogenomics and how they can be combined in the process of chromosomic diagnostics and research. It is stressed that no cytogenomic methods can be comprehensive on their own. The strengths and weaknesses of each method have to be considered. This is especially important in a time where the main stream of human genetics diagnostics is actively proclaiming that high throughput methods are able to replace all other established tests.

## 1. Introduction

Chromosomics is an interdisciplinary field of genomics and human genetic diagnostics that aims to study and understand the structure and function of the genome. The core idea is that only by combining all “traditional” (cyto) genetic approaches with all classical and new molecular biology technologies can real progress be made in this field [1,2]. It is certainly beneficial to gain deeper insights into DNA sequences [3], but one cannot understand a complete picture of genetics if sequence changes are not checked for possible effects on the morphological properties of chromosomes, their three-dimensional network in the cell nucleus and their effects on gene regulation [1,2]. Furthermore, many causes of genetic diseases are rare to exotic, such as epigenetic defects [4] or minute mosaics [5]. Such alterations are not easily accessible, nor is it cost-effective to capture them in the longed-for “single comprehensive genetic testing system”.

## 2. The Concept of Chromosomics

The term chromosomics was introduced by the late Prof. Uwe Claussen in 2005 [1]. According to his definition, this term is intended to draw attention to the morphological changes in chromosomes, which are essential elements of gene regulation. Chromosomics is concerned with the plasticity of chromosomes in terms of the three-dimensional positions of genes that affect cell function during the cell cycle in a developmentally and tissue-specific manner. It also addresses the species-specific differences in chromosome architecture that have been overlooked in the past. Chromosomics encompasses the study of changes in chromosome architecture, mediated by chromatin modification, that can influence the functions and lifespans of cells, tissues, organs, and individuals. It also addresses the occurrence and prevalence of chromosome gaps and breaks. Accordingly, chromosomics is an important aspect of genomic research and must be taken into account in human genetic diagnostics at the same time.

For decades, human genetics and genetic research in general were inextricably linked with microscopy as a core technique [6]. Between the 1970s and 2010s, the most important approach in human genetics has been (molecular) cytogenetics, with declining attention on the field [6,7]. At the same time, molecular genetics established itself slowly at first and then faster and faster [8]. The latter was an observation each visitor to human genetics conferences easily could make; sessions with a focus on (molecular) cytogenetics became more and more scare and finally disappeared completely. However, in research, cytogenetic and molecular genetic approaches are still considered equally important [9]; a kind of competition has been established in human genetics diagnostics. Cytogenetic approaches to the diagnosis of genetic disorders are considered outdated, too expensive, too ineffective, and too dependent on specialists [3,10,11]. There is no solid evidence for these statements in the scientific literature; rather, it seems to be the other way around, that high-throughput methods are much more costly than assumed [12]. At the same time, high-throughput methods are touted as being able to find every single genetic aberration [3,11], being more cost-efficient and not requiring expensive (chromosome) specialists [13].

Accordingly, the neologism cytogenomics was coined around the year 2000 to cover all approaches to genetic and genomic research and diagnostics, instead of continuing to speak of cytogenetic and molecular genetic approaches [14]. Even though there is no definition for this word in Wikipedia yet, the “International System for Cytogenetic Nomenclature” was renamed “International System for Cytogenomic Nomenclature” in 2016 [15], perhaps to encourage the reporting of molecular genetic results according to ISCN instead of the HGVS nomenclature (Human Genome Variation Society) [16].

The term cytogenomics is used here to encompass all cytogenetic and molecular genetic approaches that can be used to conduct studies in the field of chromosomics, as defined above. Accordingly, cytogenomics integrates traditional cytogenetics, molecular cytogenetics, traditional molecular genetics, and emerging genomic technologies. The great value of all these different types of cytogenomic approaches lies in their potential to be combined with each other. Here, we summarize traditional and newer cytogenomic approaches and provide examples of how their combination has led to chromosomic insights.

## 3. The Core Technologies of Cytogenomics

A review of cytogenomic approaches was recently provided [17]. To define the core technologies of cytogenomics, a chronological listing of the approaches included is first provided; it should be noted that the authors do not consider any of these techniques to be outdated.

Four core protocols are included under cytogenetics: (a) solid staining, (b) chromosome banding, (c) C-banding, and (d) nucleolar organizer region (NOR) staining. The most important insights gained in humans through these approaches include, for example, the chromosome theory of inheritance from 1902/1903, the determination of the correct chromosome number of 46 in 1956, and the identification of countless constitutional and acquired chromosome aberrations in the human population and in patients (summarized in [8]). Furthermore, cytogenetics was and is important for comparative genomics; without knowledge of the correct modal chromosome number of a given species and the localization of its centromeres and NORs, subsequent molecular data cannot be correctly interpreted [18].

Between 1900 and 1960, biochemical and molecular biological approaches laid the foundation for genetics. Ideas and findings such as the introduction of the word “genetics” in 1905, the one-gene-one-enzyme hypothesis (1941), the identification of the DNA structure (1953), the central dogma that genetic information is passed from DNA via RNA to proteins (1957), and the decoding of the genetic triplet code (1965) fell into this area (summarized in [8]). All the approaches used and insights gained at that time are still useful and can at least be applied in special research projects. Interestingly, biochemical genetic diagnostics also began during this period. Accordingly, the first screening programs based on dried blood samples were introduced in the 1960s [19]. Although they were challenged by mutation screening, such tests retained their added value in biochemical genetic diagnostics [20].

The basic molecular genetic approaches were developed mainly between the 1970s and 1980s. These include tests based on the restriction digestion of DNA (e.g., the RFLP approach, restriction fragment length polymorphism), blotting approaches of DNA, RNA, and proteins, such as Southern, Northern and Western blotting (1970s), Sanger sequencing (1975), DNA analyses based on repetitive elements such as microsatellite analysis (1980s), and many ideas starting with the idea of in vitro DNA amplification by polymerase chain reaction (PCR-1983), with the most elegant one being multiplex ligation-dependent probe amplification (MLPA) (summarized in [8]). These approaches have enabled, for example, detailed mapping of putative disease loci, the diagnosis of many types of single nucleotide changes and of uniparental disomy [4].

Molecular cytogenetics for human chromosomes was introduced in 1986 as fluorescence in situ hybridization (FISH). Starting with single- and dual-color FISH, it developed in the following decades into sophisticated multicolor FISH approaches that can be used in research and diagnostics. It can be interphase- or metaphase-oriented. In 1992, FISH on extended DNA fibers was even described [21]. All these FISH approaches are used for routine diagnostics of acquired and congenital chromosome changes and in chromosome evolution research (summarized in [8]). Insights into the three-dimensional structures of the interphase nucleus and the structure of chromosomes were possible [9]. In 1992, chromosome-based comparative genomic hybridization was published [22]. This approach, which is still very useful for comparative genomic studies between different species [23], has been further developed into the chromosomal microarray approach.

Modern molecular genetic approaches were established after 2000. The first was chromosomal microarray (CMA; also called array CGH), which was developed from chromosomal CGH. At the same time, next-generation sequencing (NGS) was further developed as a logical consequence of the Human Genome Project (HUGO), which needed solutions for faster processing of the thousands of megabases in a human cell. After that (since about 2010), NGS was developed to enable single-cell or long-range sequencing, but also to be used in sophisticated studies of interphase architecture (via the HiC approach). Variants of NGS are also able to detect isodisomies and copy number variants (summarized in [8]). The findings from the application of such approaches include, for example, the knowledge that there are about 300 human congenital diseases (microdeletion and microduplication syndromes) due to copy number variants [24], the description of topologically associated domains (TADs) [25] and a better understanding of chromothripsis [26].

One of the latest laboratory-based cytogenomic approaches was also introduced around 2010: it is applied to detect large and complex chromosome aberrations and is known as optical genomic mapping (OGM) (summarized in [8]). Finally, and not to be forgotten, the Next Generation Phenotyping (NGP) technique has been enabling the narrowing down of a potential clinical diagnosis based on facial photographs of patients since about 2015 [27]. This tool to support clinical geneticists in treating patients must also be mentioned because it can also have an impact on chromosome diagnostics and research.

Although this overview is intended to support a common view of available cytogenomic techniques, the following key differences between (molecular) cytogenetics and molecular genetics must be considered. Most (molecular) cytogenetic approaches are single-cell oriented and difficult to automate; most molecular genetic techniques target (DNA derived from) thousands to millions of cells, and sample processing can be automated. Therefore, the latter approaches are often summarized as high-throughput technologies. It is often erroneously claimed that high-throughput approaches are less expensive than others. However, this neglects and does not take into account the interpretation required in each individual case, regardless of the test used [12,28].

## 4. Advantages and Restrictions of Available Cytogenomic Approaches

It is preceded here that in the following the approaches “cytogenetics” and “molecular cytogenetics” (excluding CGH) are summarized as “cytogenetic approaches” (CAs), “classical molecular genetic approaches“ (cMAs) include “biochemical and molecular biological” as well as “basic molecular genetic” techniques, while the third cytogenomic group is the one mentioned under “modern molecular genetic approaches” (mMAs).

To understand the advantages and limitations of the available cytogenomic approaches, one must first emphasize their potential. Here is a list of what can be expected from the main cytogenomic approaches mentioned earlier:

- NGP can be useful in the diagnosis of congenital diseases by narrowing the number of preliminary diagnoses; accordingly, NGP can be extremely helpful in selecting the most appropriate confirmatory cytogenomic test(s) [27].
- CAs (banding cytogenetics or FISH) are best for determining modal chromosome numbers and numerical or structural aberrations, or even polyploidy. Neither the structure of a balanced or unbalanced rearrangement nor the ploidy of a cell can be comprehensively assessed by cMAs or mMAs [2].

The assessment of potentially significant heterochromatic variants is still best carried out by CAs such as FISH [29].

Simple cellular mosaics, as found in clinical cases, are best analyzed by CAs [5]. More complex mosaics, as found in tumors, are best analyzed by mMAs, as in liquid biopsies [30].

- CAs such as FISH (if a clinical syndrome is suspected) or mMAs such as CMA are best suited for characterizing congenital CNVs [24]. Both approaches can also be recommended for characterizing CNVs in acquired diseases [31].
- mMAs such as NGS approaches, but also Sanger sequencing, are the methods of choice for examining point mutations, small structural aberrations or CNVs at the base pair level [32,33].
- cMAs such as microsatellite analysis are still superior for detecting uniparental disomy (UPD). mMCs such as those based on single nucleotide polymorphisms (SNPs) miss at least 1/3 of cases with heterodisomy [34].
- Optical mapping is blind to the heterochromatic regions of the genome (own unpublished observations), as are standard next-generation sequencing approaches [35]. These regions can still only be examined with CAs such as FISH. As recently shown, satellite DNA appears to play a role in advanced solid cancers [36].

As emphasized by us and others, the existence of the “one” comprehensive approach, which can identify all possible genetic aberrations in one go [37], is principally not possible [28,38,39].

## 5. Suggestions for the Sequence Cytogenomic Approaches Can Be Applied Most Efficiently in Diagnostics

Depending on the clinical indication, different combinations of cytogenomic approaches must be considered. Accordingly, a proposal for modern genomic diagnostics for the intelligent combination of cytogenomic approaches could be as follows:

- Infertile patients undergoing genetic diagnosis should be studied by cMAs or mMAs to exclude disease-causing mutations, e.g., in genes such as *CFTR*, *AZF* or *FMR1* premutations [40]. In addition, CAs such as banding cytogenetics are indicated to exclude sex chromosome aneuploidies (even in small mosaics) or balanced translocations [40].
- Prenatal genetic diagnosis may include mMAs such as noninvasive prenatal testing (NIPT); however, the limitations of such methods must be considered. NIPT is a screening test that examines the placenta rather than the fetus and has a high rate of false positives [41]. Other screening tests, such as first trimester screening (including ultrasonography), may be easier. For invasive prenatal testing, CAs, cMAs, and mMAs may be indicated. For CMA as a first-line test, cytogenetics or FISH must be included in the testing scheme to understand the nature of the underlying unbalanced chromosomal rearrangement [42].
- Postnatal genetics is indicated if signs such as developmental delay, dysmorphisms, and/or delayed puberty are observed. Cytogenetics can be used first to find any gross genetic changes that may be causative. Subsequently, CMA should be performed. If no aberration is found, NGS approaches can be considered [28]. The use of AI-based tools such as NGP can also help to avoid costly whole genome analyses if a comparison of a patient’s facial phenotype with corresponding databases clearly indicates a chromosomal disorder, for example [27].
- In leukemia diagnostics, chromosome analysis should be the first test to be performed. FISH can complement these studies [43]. Large leukemia laboratories perform these tests together with panel diagnostics—knowledge of the chromosome constitution is still an important part of the WHO guidelines for leukemia classification [44].
- In the case of solid tumors, liquid biopsy—as an NGS-based approach—has already demonstrated its capabilities [33]. Nevertheless, panel diagnostics of solid tumor samples and FISH analysis of FFPE sections are equally important cytogenomic approaches in this diagnostic field [45].

## 6. Suggestions for the Sequence Cytogenomic Approaches Can Be Applied Most Efficiently in Research

In research environments of non-human genetics, there are usually not as many prejudices against long-standing approaches as in the diagnostic area anyway. Here, the scientific question is the main focus. It is obvious that it is necessary to combine CAs with cMAs and mMAs to characterize the genomes of species, compare them, and come to the correct conclusions [9,18,23,26]. This idea is also reinforced by the recently suggested concept of chromosome coding or karyotype coding [46].

## 7. Conclusions

Instead of writing our own concluding section, we prefer to finalize this review by citing Prof. Yassmine Akkari [38], as we completely agree with her well set words; she wrote: “*For decades, cytogenetics has been declared dead. But for as long as I can remember, I have never understood this statement. In my mind, cytogenetics is the science of chromosomes—and how can a science die?*” She further highlights, that cytogenetics is defined as “*a branch of genetics concerned with how the chromosomes relate to cell behavior, particularly during mitosis and meiosis*” and that “*nowhere in these definitions it is implied that this area of genetics is linked to a particular technology or that it has a limited lifetime*”. She stresses that “*the concern over the ‘longevity’ of cytogenetics lies in the fact that genetics has progressed into genomics, and by focusing on sequence variation and advanced sequencing technologies, we have neglected the principle of chromosome science and the importance of understanding chromosome behavior*.” Why was a problem made out of that? “*Because it appears that looking at a G-banded karyogram is an archaic practice and doesn’t allow for the single-nucleotide resolution that is afforded by advanced molecular methods. The real question is: are all human diseases driven by single nucleotide variation? The answer is no. Is G-banding a technology that allows for a well-established view of the genome at a single-cell level? The answer is yes. So, what’s the problem? The problem is that we didn’t fight to continue education on the science of cytogenetics*”. So, “*we started experiencing a shortage in professionals who had expertise in cytogenetics and witnessed the decrease in national programs dedicated to cytogenetics training. This, along with the rumor that cytogenetics was dead, discouraged younger generations from receiving training in the field, further accentuating the lack of innovation and appreciation. Ironically, despite a decrease in the number of cytogenetic trainees (both at the director and technologist level), the workload in cytogenetic laboratories never wavered*”. This was also highlighted by others [47,48]. “*Slowly but surely, we have started to realize that a good molecular geneticist needs to fully understand chromosome structure and function. (…) It is crucial to understand and schematically visualize the preceding meiotic event. Why? Because it will have a profound impact on our ability to provide accurate determination of recurrence risk. (…) In conclusion, cytogenetics remains extremely important, and reciprocal training and education on both ends of the DNA technology spectrum (whole chromosomes to methylation and single nucleotide aberrations) will allow true breakthroughs in genomic science*”. Or in other words: we need a concert of cytogenomic methods in chromosomic research and diagnostics.

## Data Availability

All data are available in this paper.
